# Novel insights on genetics and epigenetics as clinical targets for paediatric astrocytoma

**DOI:** 10.1002/ctm2.1560

**Published:** 2024-02-01

**Authors:** Dona A. Johns, Richard J. Williams, Craig M. Smith, Pavani P. Nadaminti, Rasika M. Samarasinghe

**Affiliations:** ^1^ School of Medicine, Deakin University Geelong Victoria Australia; ^2^ Institute for Mental and Physical Health and Clinical Translation, School of Medicine, Deakin University Geelong Victoria Australia; ^3^ The Graeme Clark Institute, The University of Melbourne Melbourne VIC Australia; ^4^ School of Agriculture, Food and Ecosystem Sciences, Faculty of Science, The University of Melbourne, Parkville Melbourne Victoria Australia

**Keywords:** epigenetics, gliomas, methylation, paediatric astrocytoma

## Abstract

Paediatric and adult astrocytomas are notably different, where clinical treatments used for adults are not as effective on children with the same form of cancer and these treatments lead to adverse long‐term health concerns. Integrative omics‐based studies have shown the pathology and fundamental molecular characteristics differ significantly and cannot be extrapolated from the more widely studied adult disease. Recent clinical advances in our understanding of paediatric astrocytomas, with the aid of next‐generation sequencing and epigenome‐wide profiling, have led to the identification of key canonical mutations that vary based on the tumour location and age of onset. These driver mutations, in particular the identification of the recurrent histone *H3* mutations in high‐grade tumours, have confirmed the important role epigenetic dysregulations play in cancer progression. This review summarises the current updates of the classification, epidemiology, pathogenesis and clinical management of paediatric astrocytoma based on their grades and the ongoing clinical trials. It also provides novel insights on genetic and epigenetic alterations as diagnostic biomarkers, highlighting the potential of targeting these pathways as therapeutics for this devastating childhood cancer.

## Graphical abstract

- Alterations in genetics, particularly epigenetic biomarkers provide significant insights into the aetiology, diagnosis, prognosis and therapeutic strategies for paediatric astrocytomas.
- Recurrent DNA methylation and histone mutations are hallmarks of astrocytoma oncogenesis in paediatrics.
- Clinically targeting these markers have shown efficacy in preclinical and clinical trials, providing new treatment options for paediatric cancer patients.

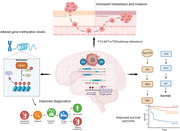

## 1 INTRODUCTION

Astrocytomas are the most common and lethal forms of glioma that affect children. They arise from star‐shaped cells called astrocytes or astrocytic glial cells and can develop anywhere in the brain and spinal cord. Among the different brain and central nervous system (CNS) tumours, paediatric astrocytomas (pA) account for 40%−50% of these, making them one of the most diagnosed solid tumours in children.^1^ Previously, the World Health Organization (WHO) classified pA into four main grades based on nuclear atypia, cellularity, aggressive/invasive nature and ability to respond to treatments (Table 1).^2^ Grade 1 pA is the most benign and generic form of pA and includes the subtypes juvenile pilocytic astrocytoma, subependymal giant cell astrocytoma and angiocentric glioma. Grade 2 pA, while remaining benign, is of a fibrillary composition capable of invasion into the healthy brain parenchyma and bordering on malignant transformation. Grade 2 pA includes subtypes of diffuse astrocytoma, pleomorphic xanthoastrocytoma (PXA) and choroid glioma of the third ventricle.^3^ Grade 3 pA are much more invasive with nuclear atypia and increased mitotic activity. These include two subtypes, anaplastic astrocytoma (AA) and anaplastic PXA. Grade 4 pA exhibits the invasiveness and recurrence of grade 3 pA, but also presents extensive microvasculature and metastatic characteristics, making these tumours one of the deadliest malignant brain tumours in children. These tumours include subtypes of glioblastoma (GBM), diffuse midline glioma (DMG) and diffuse intrinsic pontine gliomas (DIPG).^4^ Most reported cases of pA are low grade (grades 1 and 2) and account for more than 85% of all reported cases, whereas grades 3 and 4 constitute the high grade, which account to 12%−15% of all reported cases of pA. Although the 5‐year survival rate for low‐grade pA is favourable ranging between 80% and 97%, the median survival for high‐grade pA is devastatingly low with a survival rate of less than 20%.^5^ However, this classification has been replaced with a new system based on the recent ‘Blue Book’ (the WHO classification of CNS tumours has traditionally been published in a blue cover hence the name). The recently published fifth edition has moved away from the traditional classification system based on histological parameters to a more coherent classification system based on molecular markers.^6^ Table 1 shows the previous classification system of pA and Table 2 highlights the newer classification system.

**TABLE 1:** Different grades, subtypes, survival rates and clinical management of paediatric astrocytoma (based on 2016 World Health Organization classifications).^2^

					Current clinical management	
Grade	Prevalent group	Molecular subtypes	Major location	5‐Year survival rate	Treatment modality	Comments
1^7^	Ages 0−16 years	Juvenile pilocytic astrocytoma Subependymal giant cell astrocytoma	Cerebellum, brainstem, optic pathways, cerebral hemispheres of younger adults	95%–97%	Observation without intervention	
					Surgery	Most successful PFS and OS
					Early and/or radiation therapy	PFS improves but not OS
					Chemotherapy Carboplatin with or without vincristine Thioguanine, procarbazine, lomustine and vincristine	Recommended in progressive tumours upon failure of first‐line therapy
2^7^, ^8^	Younger adults	Diffuse astrocytoma Pleomorphic xanthoastrocytoma	Cerebrum, brain stem, optic pathways	78%–82%	Surgery	Awake surgery with brain mapping
					Early and/or late radiation therapy	Preferred only if surgery and chemotherapy fail
					Chemotherapy ‐ Similar to Grade 1 regimen	Second‐line therapy. Recommended when recurrence occurs or incomplete resection
3^7^, ^9^	Adults	Anaplastic astrocytoma Anaplastic pleomorphic xanthoastrocytoma	Cerebrum, cerebellum, spine and brainstem	21%–29%	Surgery	First‐line therapy for newly diagnosed progressive tumours
					Observation after surgery	
					Chemotherapy ‐ Craboplatin ‐ Lomustine, vincristine and prednisone ‐ Temozolomide	
					Targeted therapy ‐ mTOR inhibitors (everolimus)	
4^5^, ^8^	Adults except DIPG only in children	Glioblastoma DIPG	Cerebrum, spine and cerebellum	17%–28%	Surgery	Not considered a standard procedure
					High‐dose chemotherapy with stem cell transplant	Considered in recurrent tumours
					Radiation therapy	Recommended for patients without prior history of irradiation
					Targeted therapy ‐ *BRAF* inhibitor (vemurafenib)	Recurrent gliomas with *BRAF* V600E mutations

Abbreviations: *BRAF*, v‐raf murine sarcoma viral oncogene homologue B1; DIPG, diffuse intrinsic pontine/midline glioma; OS, overall survival; PFS, progression‐free survival.

**TABLE 2:** The current classification system of different paediatric astrocytoma (World Health Organization classification—fifth edition 2021).^6^, ^10^

Paediatric‐type diffuse low‐grade gliomas (grades 1 and 2)	Paediatric‐type diffuse high‐grade gliomas (grades 3 and 4)
Diffuse astrocytoma, *MYB*‐ or *MYBL1*‐altered	Diffuse midline glioma, *H3 K27*‐altered
Angiocentric glioma	Diffuse hemispheric glioma, *H3 G34*‐mutant
Polymorphous low‐grade neuroepithelial tumour of the young (PLNTY)	Diffuse hemispheric glioma, *H3 G34*‐mutant
Diffuse low‐grade glioma, *MAPK* pathway‐altered	Infant‐type hemispheric glioma

Abbreviation: *MAPK*, mitogen‐activated protein kinases.

The newer system of pA classification (WHO classification of CNS tumours fifth edition) is based mainly on the molecular details associated with each tumour. This system disregarded the Roman numeral grading system followed previously and replaced terms such as ‘entity’ with ‘type’ and ‘variant’ with ‘subtype’. One of the most notable features in this newer system of classification is the inclusion of multiple grades within single tumour types. For example, unlike the previous system where grade 1 tumours solely meant lowest severity and grade 4 tumours meant highest severity and each such grade had multiple tumour entities within them, the newer system has different grades (represented with Arabic numerals such as 1, 2, 3 and 4) for a single tumour entity. For example, PXA would have cases of grade 1 and 2 tumours as well as grade 3 and 4 tumours. Again, AA which is a grade 3 tumour is no longer called by that name instead it is labelled astrocytoma, isocitrate dehydrogenase (*IDH*) mutant, CNS WHO grade 3. This system of classification however instigates a heterogenous classification and layered report system which may confuse lay people, nevertheless this system helps clinicians with better diagnosis and individualised treatment. Despite all the new changes, a recent survey suggests that further cross‐talks between expert among neurooncologists and neuropathologists is important to advance the classification further and to ‘ensure biological relevance and clinical impact’.^10^ Table 2 lists the recent system of classification of pA according to the Blue Book.

The way an astrocytoma reacts to treatment depends on the type of the tumour and its genetic predisposition. Over the past decade, several studies have utilised integrative omics analyses using large‐scale sequencing datasets, to not only show that the pathology and fundamental molecular characteristics between paediatric and adult astrocytomas differ substantially (Table 3), but also that the molecular and genetic, including epigenetic, variations between the different types of pA can significantly alter their response to treatments. Furthermore, due to the underdeveloped immune system in paediatric patients, the effects of conventional treatments such as chemotherapy, radiotherapy and surgery, have long‐term neuropathological consequences leading to lifelong issues.^4^

**TABLE 3:** Differential characteristics of adult versus paediatric astrocytomas.^5^, ^7^

Characteristics	Paediatric and adolescent	Adult
Age group affected	0–18 years old	19–elderly
Symptoms	Headaches, behaviour changes, nausea, diplopia and papilledema	Memory loss, confusion, seizures, cognitive impairments and headache
Treatment approaches	Gross total resection followed by focal radiation	Surgery, chemotherapy using temozolomide, radiotherapy
Molecular mutation landscape	*CDKN2A/B*, *H3F3A*, *IDH*, *MYCN*, *PDGFRA*, *PTEN*	*BRAF*, *EGFR*, *H3F3A*, *IDH*, *p.K27M*, *NF1*, *TERT*, *TP53*

Abbreviations: *BRAF*, v‐raf murine sarcoma viral oncogene homologue B1; *CDKN*, cyclin‐dependent kinase inhibitor; *EGFR*, epidermal growth factor receptor; *H3F3A*, *H3* histone, family 3A; *IDH*, isocitrate dehydrogenase; *NF1*, neurofibromatosis type 1; *PDGFRA*, platelet‐derived growth factor receptor alpha; *PTEN*, phosphatase and tensin homologue; *TERT*, telomerase reverse transcriptase; *TP53*, tumour protein 53.

Precision medicine is revolutionising the way patients are treated. Using innovative animal models and integrated genomic sequencing, identifying targeted therapies that are unique to individual patients, that provide better prognosis and improved survival rates with minimal chance of relapse is on the horizon.^11^ However, the speed and extent of the translation of laboratory research to patient clinical management and therapy has been limited. In this review, we provide an update on the molecular and epigenetic alterations that characterise pA and discuss novel avenues in epigenetics for prognosis and targeted therapeutics that can be translated for the clinical management of astrocytomas in children.

## 2 EPIDEMIOLOGY AND PATHOPHYSIOLOGY OF PAEDIATRIC ASTROCYTOMAS

**FIGURE 1:**
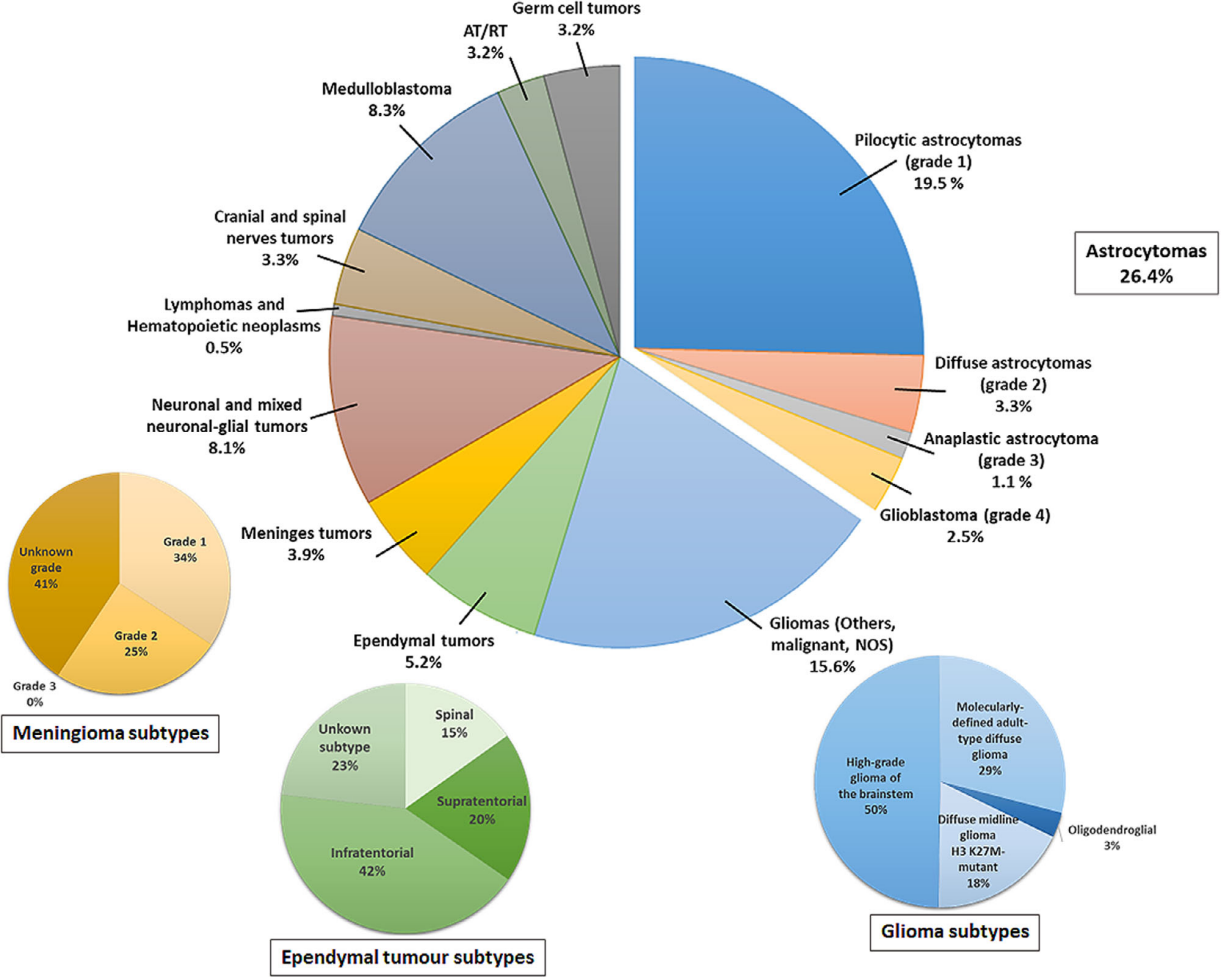
Incidence rates of brain and other central nervous system (CNS) tumours in children aged 0−14 years (2016–2020, average annual cases = 3427). All tumours and subtypes/grades (meninges, ependymal and gliomas) are based on the 2016 World Health Organization (WHO) classification of tumours of the CNS described in the 25th and 26th Central Brain Tumor Registry of the United States (CBTRUS) statistical report.^12^, ^14^, ^15^ NOS, not otherwise specified.

Tumours of the brain and CNS are the most common childhood tumours in ages 0−14 years with an incidence of 5.96 per 100 000 population, as compared to some of the other leading cancers including leukaemia (5.06 per 100 000 population) and soft tissue tumours (1.04 per 100 000 population)^1^, ^12^ (Figure 1). According to the Central Brain Tumor Registry of the United States (CBTRUS), which is the largest population‐based registry of primary and CNS‐based tumours within the whole US population, the annual incidence of pA is approximately 14 new cases per million children aged 0−14 years, with astrocytic tumours (grades 1 and 2) accounting for 40%–50% of these CNS tumours and paediatric‐type diffuse high‐grade gliomas (pHGG) (grades 3 and 4) accounting to 15%−20%.^1^, ^7^ As per the CBTRUS report, incidences of low‐grade astrocytic tumours are frequent in the early years of development (0–4 years) with an incidence rate of approximately 1.49 per 100 000 population, which reduces to 1.09 per 100 000 in ages 10−14 years. Conversely, the incidence of pHGG seems to increase with age, where approximately .16 per 100 000 population are of ages 0−4 years increasing to a rate of .29 per 100 000 at age 10−14 years. Even though these reports are limited to data available within the United States, it is still regarded as the second most reliable scientific report (subsequent to the Blue Book), which has estimates that can be projected globally.^1^, ^12^, ^13^

## 3 PAEDIATRIC‐TYPE DIFFUSE LOW‐GRADE ASTROCYTOMAS

**FIGURE 2:**
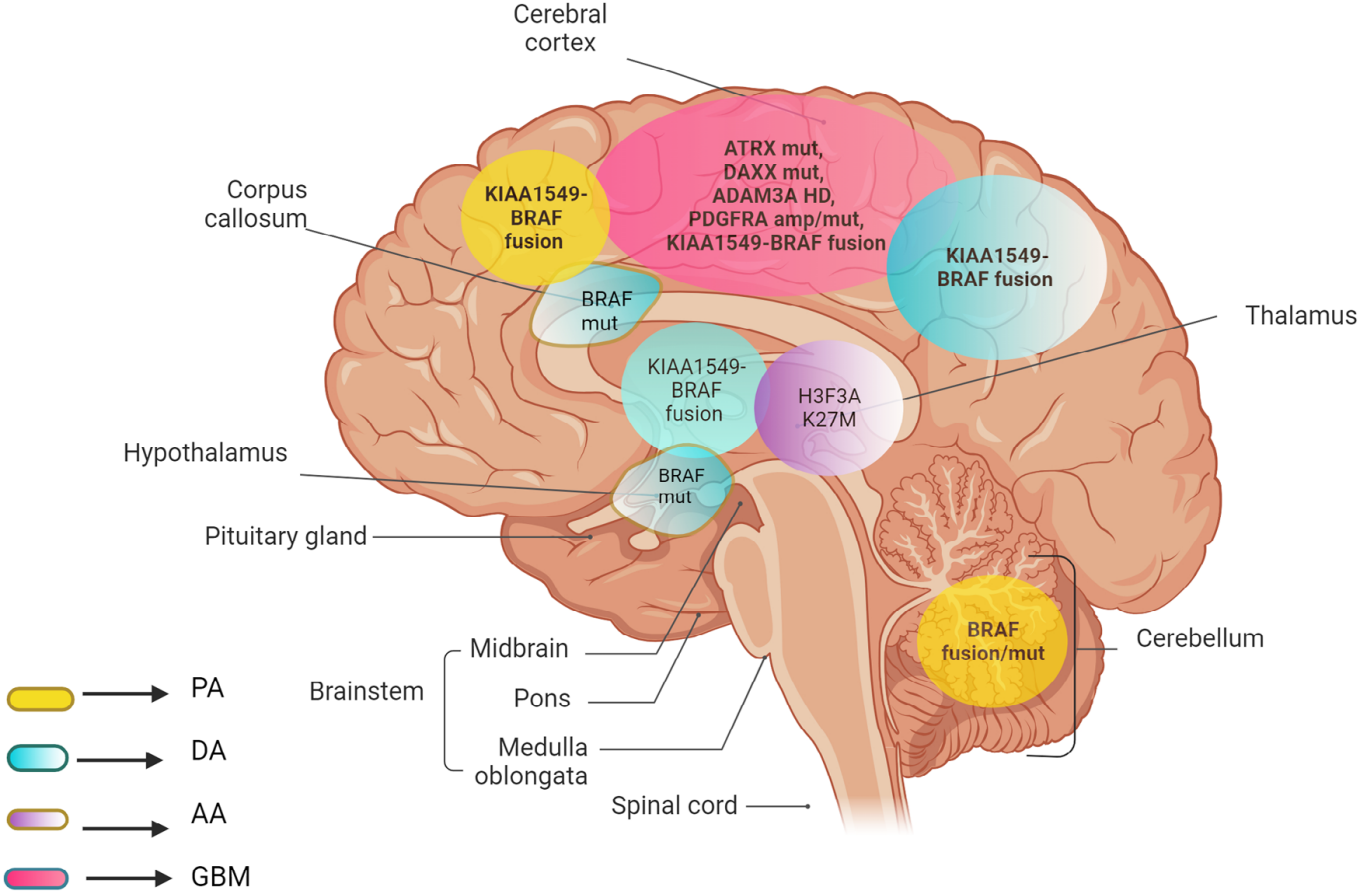
Location and primary genetic alterations of prognostic relevance in different molecular subclasses of paediatric astrocytoma (image created with Biorender.com). AA, anaplastic astrocytoma; DA, diffuse astrocytoma; GBM, glioblastoma multiforme; PA, pilocytic astrocytoma.

Grade 1 pA includes pilocytic astrocytoma, subependymal giant cell astrocytoma (SEGA) and angiocentric glioma and are the most common brain tumours in children accounting for approximately 20% of all paediatric brain and CNS neoplasms.^8^ These tumours mainly originate in the cerebellum, hypothalamic regions, cerebellum or optic nerves but can also occur anywhere within the CNS where astrocytes exist (Figure 2).^16^, ^17^ A genetic predisposition to neurofibromatosis type 1 (*NF1*) has been found to initiate the development of pilocytic astrocytomas, with 15%−20% developing tumours within the optic nerve (also known as optic glioma) before the age of 7 years. Approximately 70% of all optic nerve astrocytomas are therefore linked to *NF1* predisposition.^16^ SEGA, another grade 1 benign pA, is frequently characterised by cysts and calcification together with obstructive hydrocephalus and intertumoural haemorrhage.^18^ These tumours develop almost exclusively in children with tuberous sclerosis, which occurs in approximately 1 in 5000−10 000 newborns. Tuberous sclerosis is a multifaceted genetic disorder, which causes tumourous nodule formations in the brain, skin, eye, kidney and heart and often develops in adolescents. Around 5%−20% of patients with tuberous sclerosis have a high disposition to develop SEGA.^19^

Histopathology of grade 1 pA shows characteristic regions that are densely rich with Rosenthal fibres, consisting of cells with long hair‐like processes and hyaline structures that are highly eosinophilic, rounded, oval or beaded, with moderately irregular margins.^17^, ^20^ Due to their well‐distinguished appearance and discrete margins, these tumours are microscopically easily identifiable, however in some instances where infiltration to healthy brain regions may occur, these bipolar cells are evaluated for glial fibrillary acidic protein (GFAP) immunoreactivity to identify GFAP‐positive tumour cells. In addition, these tumours have excellent prognosis following gross total surgical resurrection and adjuvant therapies, with a 5‐year survival rate of 96% and 10‐year survival rate of 90%.^21^

Diffuse astrocytomas, *MYB*‐ or *MYBL1*‐altered, are rare tumours accounting for approximately 2%−3% of all pA. These tumours share an isomorphic entity in adults but differ in their methylation cluster profiling. These tumours behave indolent in children as well as adults and are usually associated with *MYB1*–*QK1* gene fusions. Polymorphous low‐grade neuroepithelial tumour of the young (PLNTY) is another rare and indolent tumour with a distinct DNA methylation profile and consistently harbours mitogen‐activated protein kinases (*MAPK*) pathway constituents such as v‐raf murine sarcoma viral oncogene homologue B1 (*BRAF*) proto‐oncogene. Diffuse low‐grade gliomas (LGG) *MAPK* pathway‐altered tumours are associated with almost all *MAPK* pathway‐associated anomalies, and are presented without any *IDH1/2* and *H3F3A* mutations. As all these entities have relatively impaired expressions of the *MAPK* pathway, a more robust system is needed to diagnose these entities with precision.^6^, ^10^, ^22^

Grade 2 pA, according to WHO classifications, are regarded as low‐grade tumours. As per the previous classification system, grade 2 astrocytomas comprise of two main subtypes, diffuse or fibrillary astrocytoma and PXA, which accounts for approximately 10% of all paediatric brain and CNS neoplasms.^1^, ^6^ These tumours are more aggressive and diffused in nature than grade 1 astrocytomas, with tumours exhibiting greater patterns of infiltrating tumour cells and undefined borders. In the recent system of classification, these tumours are grouped as paediatric‐type diffuse LGG, *MAPK* pathway‐altered. Grade 2 pAs predominantly originate in the supratentorial region, deep midline structures and the corticomedullary region, with diffuse pA growing more in the hypothalamic chiasmatic region and pleomorphic subtypes occurring primarily in the superficial temporal region within the cerebrum.^23^ Histologically, grade 2 pA demonstrates greater infiltration and cellularity and fewer microcysts and nuclear pleomorphism. Diffuse astrocytomas, characterised by the lack of clear separation between tumourous and normal parenchymatous brain tissues, show increased cellularity and mild nuclear atypia.^24^ These tumours are also known to harbour mutations in the gene *IDH 1* or *IDH 2*, which has led to those diffuse astrocytomas without *IDH* mutations being referred to as *IDH* wild‐type, also classified as a grade 4 tumour.^9^ Diffuse astrocytomas also have a grading system within the subtypes based on the degree of vascular proliferation, mitotic activity and necrosis. Generally, *IDH* wild‐type diffuse astrocytomas are the most malignant and lethal, grouping them as grade 4 tumours. Grade 2, *IDH* mutant astrocytomas are well‐differentiated tumours with low mitotic activity, and no necrosis with limited or no vascular differentiation and very rarely progress into higher grade malignant transformations.^25^

PXA, which were exclusively grade 2 tumours in the previous classification system but are now regarded as either grade 2 or 3 based on the histological features, are relatively rare in nature (less than 1% of all astrocytomas) and are usually found in younger adults. The average age of diagnosis is 12 years, with most cases originating as cortical tumours with a cystic component in the temporal lobes and extending into the leptomeninges and Virchow–Robin spaces. Along with all the other classic symptoms of low‐grade astrocytoma, PXA is associated with varying degrees of hemiparesis weakness on one side of the body and severe seizures. Microscopic analysis of PXA tissues shows variable histological features, including polygonal, spindle and multinucleated giant cells, characteristics that give its name ‘pleomorphic’.^26^

## 4 CURRENT MANAGEMENT OF PAEDIATRIC‐TYPE DIFFUSE LOW‐GRADE ASTROCYTOMAS

With low‐grade pA usually showing a favourable prognosis, clinical management of these tumours utilises a multimodal approach, which includes surgery, radiotherapy and chemotherapy. As the first‐line therapy, complete surgical resection is recommended, which has been associated with outstanding overall survival (OS) of 80%−100%.^27^ Generally, tumours of the cerebrum and cerebellum are the most acquiescent to surgical excision, however, the majority of low‐grade pA arise in locations that are problematic to resect, such as the brain stem, hypothalamus and optic chiasm, leading to partial tumour resections. Furthermore, in association with genetic predispositions such as *NF1*, tumours tend to be multifocal in origin and are increasingly more difficult to resect.^21^, ^28^ While the survival rate of patients with partial tumour resection is to some degree favourable, with an OS rate around 50%−95%, many patients do require adjuvant therapy as the risk of tumour recurrence is high. If secondary tumour progression is identified, surgery is reconsidered, however with recurrent tumours being more infiltrative and aggressive, radiation therapy provides an effective option in most cases.^24^

In patients older than 3 years of age and diagnosed with either diffuse pA or PXA, radiotherapy with 36−52 Gy photons (X‐ray beams) in 1.8 Gy fractionation is recommended.^29^ Although OS is not greatly affected by the use of radiation, early intervention with radiation soon after biopsy or surgery has shown a 5‐year progression‐free survival (PFS) of 61%−77% and OS of 90%−97%, as compared to patients that did not receive radiation post‐surgery (radiation received at disease progression), which had a PFS of 50% and OS of 84%.^30^ It should also be noted that early radiation showed no benefit in survival when partial tumour resection had occurred, and that the efficacy of radiation was only beneficial with gross total resection (GTR). Recent advances in X‐ray technology including stereotactic and convergence therapies have successfully reduced the side effects of radiation therapy.^31^ Even though it is still regarded as an important tool in the clinical management of both adult and pA, the side effects of radiation usually caused by the latter side scattering phenomenon exhibited by high‐energy X‐rays, have grave consequences on young patients. These involve significant life‐long complications including neurological, cognitive, visual and auditory deficits. The possible role that radiation is also linked to the formation of secondary malignancies, its adverse side effects and questionable survival benefit, has led to its recommendation, particularly in younger patients (<10 years old), to be considered only after all other therapy strategies have been expended.^32^

Due to the long‐term side effects associated with radiation, chemotherapy is highly regarded as the first‐line adjuvant therapy for the clinical management of pA. A recent study on OS of patients that received radiation alone or chemotherapy alone in surgically resected LGG showed that the median OS was 125.8 months for the chemotherapy only group as compared to only 98.9 months for the radiotherapy group.^33^ A similar study that looked at whether these treatment regimens given alone improved OS in patient without surgical resection showed that regardless of age, chemotherapy alone improved OS significantly as compared to radiotherapy alone.^34^ Chemotherapy is introduced after surgery, as a post‐operative treatment regimen and is aimed at inducing tumour shrinkage, and recurrences and delaying the need for radiation. For progressive low‐grade pA, the most common chemotherapy regimens used are either carboplatin + vincristine (CV)^35^ or thioguanine, procarbazine, lomustine and vincristine (TPCV).^36^ Studies have shown that the 5‐year event‐free survival rates (EFS) of CV treatment regimen was around 39% while that of TPCV was 52% (NCT00002944). Children with *NF1* showed higher tumour response rates to the CV when compared to those without a *NF1* genetic predisposition. *NF1* patients had a 5‐year EFS of 69% as compared to 39% for non‐*NF1* patients.^37^ TPCV is not considered for patients with *NF1*, due to the underlying predisposition to leukaemia and the high risk of forming secondary leukaemia with the use of lomustine and procarbazine.^38^ Other treatment regimens used for low‐grade pA include bevacizumab, everolimus, 5‐fluorouracil, irinotecan, temozolomide (TMZ), vinblastine and platinum‐based drugs such as cisplatin and etoposide.^39^, ^40^ Different combinations of these agents at different dosages and durations have been explored in clinical trials over the past decade; however, their use is associated with adverse side effects such as sepsis, myelosuppression and high morbidity and show minimum clinical benefits.

## 5 PAEDIATRIC‐TYPE DIFFUSE HIGH‐GRADE ASTROCYTOMAS

As per the previous classification, grade 3 astrocytomas include the molecular subtypes AA and anaplastic xanthoastrocytoma (AXA) (Figure 2). In contrast to its rate of incidence in adults, paediatric AAs are relatively sporadic accounting for less than 10% of all CNS tumours in children.^40^, ^41^ Anaplasia is the term used to define under differentiated cells lacking morphological features of mature/differentiated cells, and this usually occurs in cells that undergo malignant transformations.^41^ However, the latest classification system has now omitted the word ‘anaplastic’ and is now called astrocytoma, *IDH*‐mutant, CNS WHO grade 3. These rare tumours are heterogeneous and diffusive and show extensive mitoses, invading deep white matter tracts, and are mostly found near cerebral hemispheres. AA is similar in histopathology to GBM across paediatric as well as the adult population. Despite these histological resemblances, they differ significantly in their molecular characteristics and hence require specific treatment and prognostic strategies.^41^

The paediatric form of astrocytoma, *IDH*‐mutant, CNS WHO grade 3, is reported to have a 5‐year survival rate of approximately 25%. Causes for its occurrence are not completely understood although certain genetic mutations and exposure to radiation therapy have been identified as some of the contributing factors. Commonly associated symptoms of these tumours include vomiting, change in moods and mental state, lethargy, seizures and hemiplegia associated with tumours located in the frontal lobe. One of the interesting characteristics of these tumours is that they are usually associated with genetic disorders such as NF1 syndrome, tuberous sclerosis and Li–Fraumeni syndrome, which were discussed earlier in the review. It must also be noted that there are several reports of a correlation between the incidence of anaplastic tumours in either of these conditions and a high probability of these molecular alterations being genetically inherited, which does not happen otherwise.^42^, ^43^

Grade 4 astrocytomas are the most malignant of all CNS tumours. The most common grade 4 tumours include two molecular subtypes namely, GBM and DIPG, both of which exhibit extensive mitotic activity, haemorrhage, necrosis and microvascularisation.^44^ Paediatric GBM (pGBM) has a mean incidence rate of .6–.85 per 100 000 patients in United States aged 0 −19 years old.^1^ In children, primary GBMs usually originate de novo within the cerebrum, cerebellum, brain stem and spinal cord, whereas secondary GBMs originate from the malignant transformation of other LGG, in particularly diffuse and AA.^45^ pGBM has a varying range of symptoms depending upon the age and location of GBM. Some of the symptoms include hemiparesis, dysphasia, aphasia, impaired memory, headache, nausea and increased intracranial pressure. Histologically, these tumours are diffusely infiltrative, with irregular heterogeneous textures and ill‐defined boundaries. Other visual markers include multinucleated cells, sporadic nuclei, satellite lesions, neovascularisation and calcific components (mostly in secondary GBM).^46^

DIPG (now regarded as diffuse hemispheric glioma, *H3.3G34* mutant) on the other hand have a lower incidence of .05–.06 per 100 000 with a median age of diagnosis between 6 and 9 years old. DIPG is primarily found within the pons, brain stem and spinal cord and the most common clinical symptoms include motor deficits, facial weakness, dysarthria, diplopia and conjugate gaze palsy as a result of dysfunctional pontine structures.^46^ Also in the latest classification two new subtypes have been defined for DIPGs with *H3* wild‐type with loss of *H3K27* trimethylation such as DMG, EZH inhibitory protein (*EZHIP*) overexpressed and DMG, *EGFR* mutant. Histopathological features of DIPG are similar to that of pGBM with the majority of tumours showing necrotic features, microvascular proliferation and mitotic structures. Even though the histopathologic features of high‐grade gliomas (HGG) are similar in children and adults, the molecular paradigms underlying these tumours differ significantly leading to different management regimens in children as compared to adults.^46^, ^47^, ^48^

According to the recent classification changes, high‐grade tumours include DMG, *H3 K27*‐altered, diffuse hemispheric glioma, *H3 G34*‐mutant, diffuse hemispheric glioma and *H3 G34*‐mutant infant‐type hemispheric glioma. However, neurooncologists and neuropathologists are still advocating for more clarification on the classification of paediatric tumours. For example, as mentioned above in a world‐wide survey by Gielen et al. involving 187 neurooncologists and 160 neuropathologists 68% advocated for the reintroduction of paediatric subtypes of AA and GBM.^10^ Again, 63% suggested that a new group called ‘Anaplastic pilocytic astrocytoma WHO 3’ to include pilocytic astrocytoma types with anaplastic features analogous to WHO grade 3 tumours. Therefore, the following sections of this review are based on the different grades of paediatric brain tumours rather than the newly defined entities.

## 6 CURRENT MANAGEMENT OF PAEDIATRIC‐TYPE DIFFUSE HIGH‐GRADE ASTROCYTOMAS

Similar to low‐grade pA, a multimodal treatment approach including surgery, chemotherapy and radiation stipulates the current treatment regimen against pHGG, however, unlike with low‐grade tumours, these therapies remain to provide exceedingly poor prognosis and long‐term adverse effects on children that have high‐grade pA.^49^ Prognostic indicators for pGBM and DIPG are still being researched, as of now, the extent of surgical resection of a tumour is the most reliable prognostic marker of PFS and OS in pHGG.^50^, ^51^ Several studies have confirmed the importance of GTR of tumours during initial surgery as it leads to significant improvements in PFS and OS.^50^, ^52^, ^53^, ^54^ In two independently conducted cancer survival analysis studies led by Mišković and coworkers^53^ and Quiñones‐Hinojosa and coworkers,^55^ it was shown that age, race, tumour location and disease presentation had no effect on OS, however, the type of surgical removal of the tumour led to substantial differences in mortality. GTR increased mean survival time by 61.5 months as compared to subtotal resection and the mortality rate was much lower in patients who underwent GTR when compared to partial resection, biopsy only or those who did not undergo surgery. A more recent form of surgical intervention that has gained interest in managing HGG is supramaximal resection (SMR). This involves expanding the magnetic resonance imaging (MRI) T1 weighted region (usually used for GTR) further out as mapped utilising MRI T2 FLAIR method, allowing the resection of infiltrative/diffused cancerous tissue.^56^, ^57^ Depending on the physiology of the tumour and the location, a range of studies have shown that SMR significantly improves PFS and OS of patients, compared to those that underwent GTR or partial resection surgery.^58^, ^59^ Other novel surgical procedures that improves GTR and prevents surgery‐associated complications involve using intraoperative imagining such as MRI or ultrasound for real‐time visualisation of the tumour region, fluorophores such as sodium fluorescein (SF) and 5‐aminolevulinic acid (5‐ALA), dyes that bind to proteins in the blood at the tumour site (SF fluorophore) or metabolised by cancer cells (5‐ALA fluorophore), which can be detected under ultraviolent light and the use of motor evoked action potentials in real‐time to help monitor vital regions of the brain that are involved in motor functions. These innovative procedures have shown significant improvements in GTR and survival rates in adults as compared to surgery alone.^56^ Nonetheless, it is important to note that successful resection of tumours relies greatly on the location as well as its pathological extensions.^45^ DIPG, a brainstem tumour, and HGGs that arise in the optic nerves, midline structures and the spine are often challenging to resect completely and usually results in severe neurological defects.

In cases where GTR is not possible, chemotherapy is recommended over radiation. Unlike with LGGs, radiation therapy has shown dismal effect on OS in patients with HGGs. Trials on DIPG and pGBM comparing conventional fractionated (54 Gy for 5−6 weeks (1.8‐Gy daily fractions) and hypo‐fractionated radiotherapy (39/3‐Gy daily or 44.8/2.8‐Gy daily fractions) have shown that hypo‐fractionated radiation leads to fewer neurocognitive complications as a result of the treatment but showed no difference in survival outcomes and PFS as compared to conventional radiation.^60^, ^61^, ^62^ Therefore, radiation therapy is usually only recommended in patients older than 3 years of age, primarily due to harmful effects it has on the developing brain in younger children, and if surgical interventions are unsuccessful, if disease progression or tumour recurrence occurs.^63^

Numerous chemotherapies have been tested, as a single agent or in combination with other chemotherapeutics and radiation therapy and found to have poor survival benefits in children with HGGs when compared to conventional treatments.^64^ Some of the most commonly tested therapies include bevacizumab, carmustine, capecitabine, carboplatin, cisplatin, cytosine arabinoside, dimethyl‐triazenoimidazole‐carboxamide, etoposide, gemcitabine, hydroxyurea, methotrexate, procarbazine, prednisone, TMZ and vincristine; however, none has shown any therapeutic benefits on OS and in most cases results in significant drug‐induced toxicities.^65^

TMZ is regarded as the ‘drug of choice’ for treating adult GBM, but it is equitably inefficient in pGBM. Due to the varying molecular and histological signatures of paediatric and adult HGG, it is nearly impossible to adapt treatment modalities interchangeably.^66^ This was proved in a study by Narayana et al., which reported the use of TMZ and bevacizumab in adults and paediatrics with recurrent HGG and showed that children responded poorer to the treatments as compared to adults. pHGG patient's median OS and PFS were approximately 2.9 and 3.2 months lower than in adults that were given the same regimen.^67^ This trial together with other similar clinical studies that have looked at TMZ as a single agent or given in combination with radiation therapy or with adjuvants such as Lomustine and methylguanine methyltransferase (MGMT) inhibitors have been unsuccessful,^65^ which has significantly hindered the use of TMZ or any one chemotherapeutic agent as an accepted universal standard therapy. In a survey conducted to determine the most recommended standard of care by paediatric oncologists and neuro‐oncologists in treating patients with HGGs, 30.7% recommended the protocol as per The Children's Oncology Group (COG) ACNS0126 trial, which involved concomitant TMZ with RT followed by TMZ^68^, ^69^ and 16% would recommend radiation with TMZ and bevacizumab followed by maintenance with TMZ or bevacizumab as per the ACNS0822 protocol.^50^ Interestingly, 40% of clinicians would suggest regimens depending on recent advances in the literature and the patient's health status such as their genetic profile, toxicity, ability to tolerate medications and their quality of life.

In summary, clinical management of both low‐grade and high‐grade paediatric/adult astrocytoma is governed by four key prognostic factors, age at diagnosis, MGMT methylation status, level of surgical intervention and use of chemotherapeutics such as TMZ. Other factors associated with increased prevalence and mortality rates in different regions and communities include socio‐demographic and economic factors. These include race, where Whites/Caucasian children and adolescents show higher incidence rates for CNS tumours as compared to Black, American Indian and Asian/Pacific islanders however survival outcomes are significantly poorer in these races as compared to Whites.^14^ Parent's education, cultural views, access to healthcare and family income are some of the other factors linked to poor survival in children from different ethnic backgrounds. Most children that lived in socially deprived regions had higher death rates than those that lived in socially affluent areas and it was reported that lack of access to the healthcare system, to specialised health care providers and to different clinical management regimens had a profound consequence on the survival of paediatric cancer patients.^8^, ^70^, ^71^, ^72^

## 7 GENETIC ALTERATIONS OF CLINICAL RELEVANCE IN PAEDIATRIC ASTROCYTOMA

**FIGURE 3:**
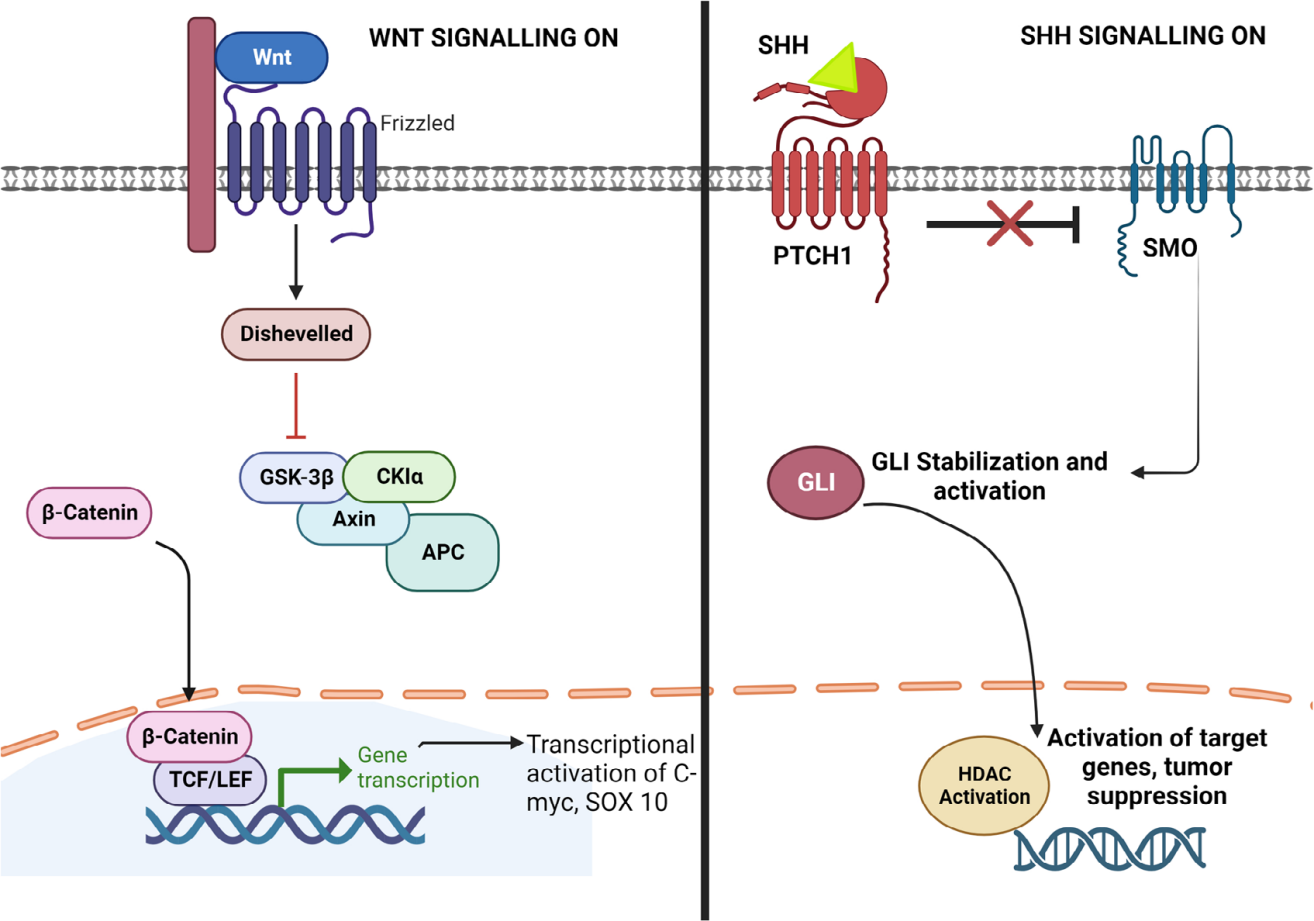
Schematic of the wingless‐related integration sites (WNT) and sonic hedgehog (SHH)‐based molecular signalling pathways across paediatric astrocytoma. Unphosphorylated β‐catenin localises in the nucleus and activates transcription of genes such as C‐MYC, SOX10 and cyclins. In the absence of SHH ligand, smoothened receptor (SMO) is inhibited by protein patched homologue (PTCH), leading to the formation of glioma‐associated oncogene homologue 1 (GLI) repressor that inhibits target genes. SHH ligand interacts with PTCH resulting in a signalling cascade which activates transcription of genes associated with tumour proliferation (image created with Biorender.com). APC, adenomatous polyposis coli; CKIα, casein kinase 1‐alpha; GSK‐3β, glycogen synthase kinase 3 beta; TCF/LEF, T‐cell factor/lymphoid enhancer factor.

Molecular alterations occurring in pA can be grouped into those arising in paediatric‐type diffuse low‐grade gliomas (pLGG) and those occurring in pHGG. Recent advances in omics technologies have made it easier to elucidate gene–gene and gene–environment interactions to better understand how they contribute to mechanisms aiding the tumourigenesis of pA. Such molecular alterations are usually identified via techniques such as immunohistochemistry,^73^ fluorescent in situ hybridisation, next‐generation sequencing, methylation assay, SNP assays, droplet digital PCR and nanostring counters.^74^ Not only have these techniques established that there are molecular differences between low‐ and high‐grade tumours, but there are also key molecular differences between paediatric and adult astrocytomas, warranting the need for personalised therapies.^75^, ^76^ According to current literature, the major pathways responsible for the metastatic progression and malignant transformation of brain cancers, in general, are *RAS*–*MAPK* pathway, janus kinase–signal transducers and activators of transcription (*JAK*–*STAT*) pathway, phosphatidyl inositol‐3 kinase–Akt (*P13K*–*Akt*) pathway, sonic hedgehog (*SHH*) pathway and wingless‐related integration sites (*WNT*) pathways (Figure 3). Of these *WNT* and *MAPK* pathways are the most active in tumours of astrocyte origin and in paediatric astrocytic tumours, with *MAPK* being the most studied pathways.^77^

One of the notable differences in paediatric and adult astrocytomas is *IDH 1/2* mutations. These genes are frequently and universally mutated in adult tumours in which mutations commonly occur in approximately 70% of grade 1, grade 2 and secondary neoplasms,^78^ however, in paediatrics only 0%−17% of cases are reported to harbour *IDH 1/2* mutations, with common occurrences in adolescents and young adults.^79^, ^80^ Another age‐associated genetic difference is the absence or very rare frequency of *EGFR* mutations in children as compared to adult tumours. According to Nakamura et al., *EGFR* mutations commonly reported in adults were rarely observed in paediatric tumours, where no *EGFR* amplification was observed in pLGG and only 10% expression was observed in pHGG.^81^ This study corroborates with the study by Wasson et al., which reported similar results and also suggested that astrocytomas in children aged 6 years and under differ from those aged above 6 years,^82^ therefore, implying that independent research needs to be done for different age groups of children to formulate specific treatment regimens based on their molecular characteristics. Other molecular differences between paediatric and adult astrocytomas include the increased frequency of *ACVR1*, *ATRX*, *H3F3A*, *KIAA1549‐BRAF*, fibroblast growth factor receptor 1 (*FGFR1*) mutation and fusions, SET domain‐containing 2 (*SETD2*),^83^ *PDGFR‐α* and *P53* mutations in paediatric tumours compared to relatively low to no mutations in adults.^75^, ^76^

## 8 COPY NUMBER VARIATIONS OF CLINICAL RELEVANCE IN PAEDIATRIC ASTROCYTOMA

Another genetic change commonly seen in astrocytomas is known as copy number variations (CNVs), which is a type of chromosomal abnormality. CNV refers to the phenomenon where the number of copies of a DNA segment varies among genomes. Such variations could be short or long and can result in varying physiological traits as well as contributing to rare genetic disorders. Since the introduction of the 2016 version of the WHO classification system, CNV and their potential role in the pathogenesis of astrocytomas has been a topic of growing interest.^84^ Chromosome shattering or ‘chromothripsis’ has been regarded as the most common mechanism that leads to CNVs in localised tumours. CNVs associated with *IDH1/2* gene mutations is a robust prognostic factor in most adult and pA histologies and is therefore extensively tested in pHGG.^85^ In 2015, Cohen et al. demonstrated that the number of CNVs in *IDH* mutant GBM is significantly higher than that seen in LGG. Several other studies have established a strong correlation between CNV and prognosis in pA, especially HGG.^85^ For example, *IDH* mutant GBM in children and adults have a better prognosis compared to *IDH* wild‐type tumours. Also, LGG's harbouring *EGFR* amplification CNV's such as + 7 and −10, and mutations in genes such as *TERT*, *NKX2.2* and *EGFR* have aggressive clinical features and low survival outcomes mimicking the features of a HGG.^86^, ^87^ PA usually exhibit +1q, −11q and −16q as well as +4q, and generally harbour considerably fewer CNVs than adults.^88^ A study looking at the correlation of contributing factors of mutational burden in LGG and HGG showed that CNVs and loss of heterozygosity (LOH) clustered together, distinct from molecular alterations, suggesting that these changes may have a common lineage.^89^ Research on CNVs are hence much needed to further our understanding of these alterations and the clinical impact they have on the pathogenesis of pA.

## 9 RECENT CLINICAL ADVANCES TARGETING GENETIC ALTERATIONS

**FIGURE 4:**
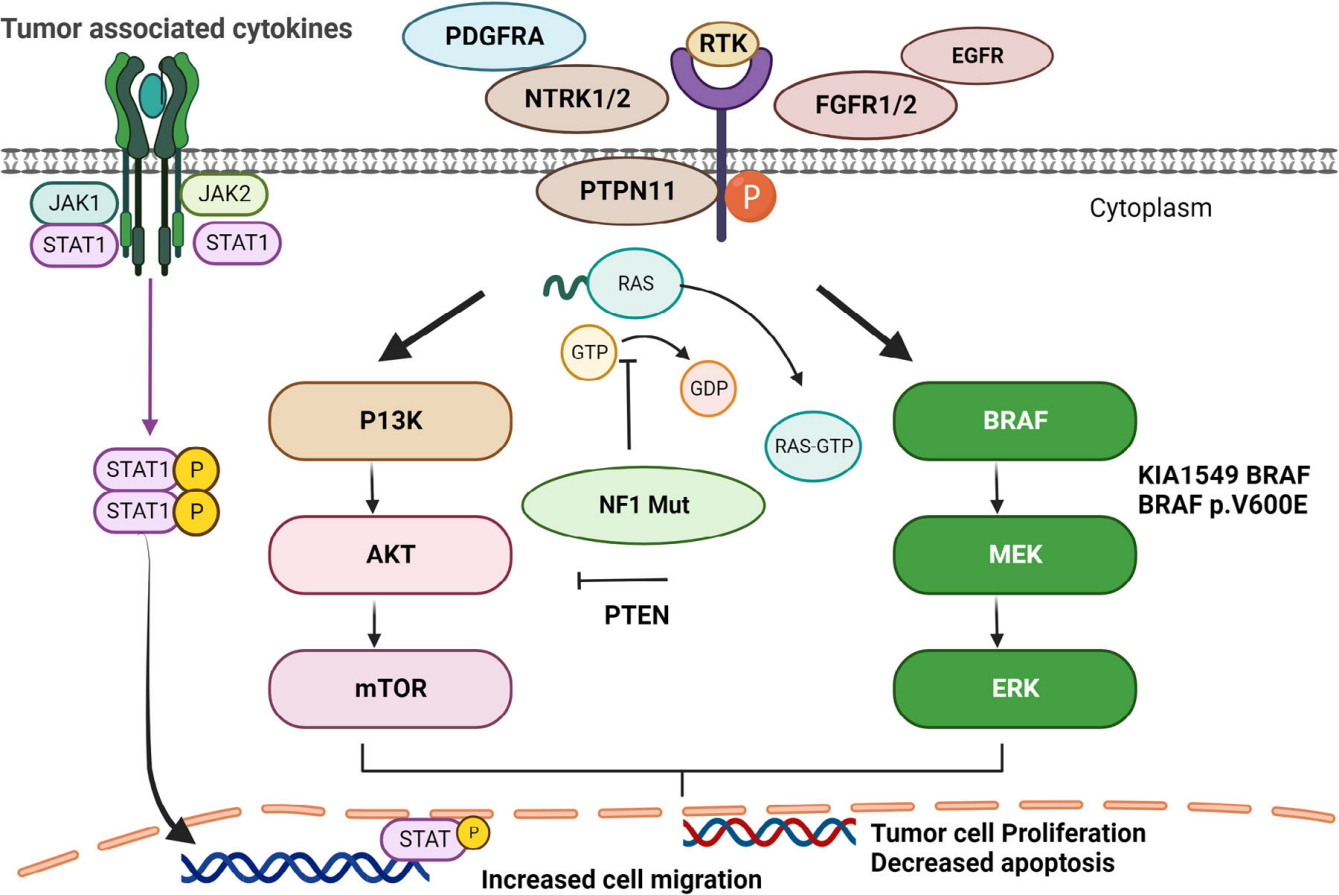
Schematic of the RAS/MAPK and JAK/STAT‐based molecular signalling pathways altered in paediatric astrocytoma (image created with Biorender.com). GDP, guanosine‐5′‐diphosphate; GTP, guanosine‐5′‐triphosphate; JAK, janus kinase; MAPK, mitogen‐activated protein kinases; STAT, signal transducers and activators of transcription.

Until almost a decade ago, it was unknown what key genetic alterations caused the initiation and oncogenesis of pLGG, however recent advances into understanding the underlying molecular mechanisms has led to the knowledge that almost all pLGG harbour genetic alterations that promote the upregulation of *RAS/MAPK* pathway.^17^, ^24^ Some of the major alterations identified in this pathways are associated with *BRAF* (∼52% frequency), with *KIAA1549:BRAF* gene fusions approximately 48% and *BRAF* ^V600E^ around 8%,^90^ *NF1* (15%), *FGFR1* (∼12%), protein tyrosine phosphatase non‐receptor type 11 (*PTPN11*) (<2%) and neurotrophic receptor tyrosine kinase 2 (*NTRK2*) (<1%) fusion genes (Figure 4). In addition, other more secondary, non‐*MAPK*‐related genetic alterations that occur at a much lower frequency yet is reported to play a role in the development of LG pA are Myb proto‐oncogene protein (*c‐MYB*), tuberous sclerosis complex 1/2 (*TSC1/2*), *IDH1*, *H3F3A* and cyclin‐dependent kinase inhibitor 2A (*CDKN2A*).^20^

Currently, most clinical trials and research are focused on targeting molecules that can act as potential inhibitors of the *MAPK/ERK* pathway. This pathway has components that are expressed in normal brain environment, and they are involved in different neurological functions such as cortical neurogenesis, development of the cerebellum and perception of pain and memory. Studies have shown increased activation of *BRAF*–*KIAA* fusion, in the cerebellar and optic pathways in tumour progression in paediatric patients^91^ and functional inactivation of *MAPK/ERK* components, facilitates the neuronal progression of astrocytic molecular markers such as *SOX10*, platelet‐derived growth factor (*PDGFα*) receptors and neural/glial antigen 2 (*NG2*) proteoglycans.^92^ The influence of these mutations on *MAPK* signalling pathways have innovated multiple trials that look at targeting *BRAF* activity using inhibitors such as vemurafenib (NCT01748149) and dabrafenib (NCT01677741). Safety and efficacy profiles of these drugs used as monotherapy reported them to be well tolerated in patients with pA with manageable toxicity even at maximum exposure levels and had anti‐tumour activities.^93^, ^94^, ^95^ This has led to several phase 1/2 clinical trials evaluating combination therapies. These include dabrafenib used in combination with trametinib (MEK 1/2 inhibitor) in patients with pLGG (NCT02124772) and pHGG (NCT02684058) and on patient that have undergone radiation therapy (NCT03919071). While the latter is still in the recruiting stage, results from the other combination therapy trials have shown promising therapeutic outcomes. In patients with pLGG, combination therapy was well tolerated than monotherapy and the PFS was 36.9 months with dabrafenib and trametinib as compared to 16.4 months with trametinib given alone.^96^ Similarly, in pHGG patients, combination therapy was found to have higher overall response (56%) and complete response (29%) as compared to dabrafenib alone (45% and 10%).^97^ These first‐generation *BRAF* inhibitors have shown durable response rates and stable conditions in pA indicating their potential therapeutic efficacy.^95^ However, these inhibitors cause paradoxical activation of *MAPK* pathways by mediating *RAF*‐independent activation of *MEK* and *ERK* when used against tumours that are BRAF wild‐type or KIAA1549‐BRAF fused and can cause increased tumour progression and growth.^98^ Therefore, a new line of BRAF inhibitors, also called second‐generation paradox‐breakers, are currently being tested as alternative therapies against several adult cancers but is yet to be tested in paediatrics.^99^

*NF1*‐associated mutations are the most studied mutation in low grade tumours and it is one of the most studied through clinical trials. For example, the effects of an immunotherapy called Lenalidomide is currently being evaluated on paediatric patients with a predisposition to *NF1*, with recurrent pilocytic astrocytomas or optic nerve pathway gliomas to determine the best dose–response of the drug (NCT01553149). Results of this study are yet to be analysed. Another clinical trial that used selumetinib (*MEK 1/2* inhibitor) on children with and without *NF1* deficiency identified a higher partial response (40%) and 2‐year PFS (96%) in patient with a *NF1* predisposition and compared to those without a *NF1* deficiency (NCT01089101).^100^ Other targeted therapies studied in pLGG include larotrectinib (NCT02637687, NCT02576431)^101^, ^102^ and entrectinib (NCT02650401).^103^ These drugs target tropomyosin receptor kinases including *TRKA/B/C*, *ROS1* and *ALK* fusion mutations. Promisingly, safety and efficacy trials on paediatric patients were well tolerated and had therapeutic anti‐tumour effects. These drugs are currently in phase II trials to further evaluate their therapeutic potential. All these clinical trials signify the importance of targeting molecular marker aided therapeutic regimens in combating pLGG.

Unlike pLGG, research into finding key genetic alterations in pHGG has seen significant advances in recent years. It has been established that somatic histone coding gene mutations in *H3* and *H3.1* occur at high frequency in pHGG such as AA and GBM^104^ and that these alterations are linked to two of the most researched cancer predisposition symptoms of pHGG namely *NF1* and tuberous sclerosis type complex. Some of the most studied genetic alterations in pHGG are associated with the tumour suppressor pathways involving *TP53*, *NF1*, *EGFR* and *RB1* genes. Global gene expression and copy number assays have also shown that mutations occur universally in *IDH1* genes as a proneural signature, which is considered as one of the epigenetic clusters in HGG.^105^

However along with genetic alterations such as these, pA have been known to have extensive chromosomal imbalances such as loss or gain of short or long arms. For example in one study by Rickert et al., 10 out of 10 AA samples and 11 out of 13 GBM samples consisted of chromosome gain/loss, with +1q occurring with the highest frequency (54%) followed by −11q (22%) and −22q (45%).^88^ An example of such structural alterations affecting gene expression is the *H3.3* (*H3F3A*) mutation at glycine 34, which occurs exclusively with *ATRX* and *TP53* mutations. According to MacKay et al.,^106^ tumours that exhibit *H3.3G34* alteration is the only pHGG subtype that exhibits *MGMT* methylation rates exceeding 20%, which has exclusive effects in predicting the prognosis of pHGG. PXA‐like molecular alterations are another characteristic genetic alteration in these tumours, which harbour *BRAFV600E* mutations shown to favour *MGMT* hypermethylation. These changes accumulate over the course of epigenetic evolution and reflect the genetic profile of some other genes such as *KIAA1549–BRAF* (seen in pLGG), *VEGF* and *ATRX*.^107^

Over the past couple of decades, several different clinical trials have looked at the effects of various combinations of chemotherapies in conjunction with radiotherapy and surgery as treatment regimens to improve survival and quality of life of children with high‐grade astrocytomas. To date, the accepted regimen for pHGG involve complete/maximal surgical resection of tumour followed by radiotherapy (if older than 3 years of age) and treatment with bevacizumab and/or TMZ.^108^ Several randomised trials are currently looking at the effects of carboplatin, thioguanine, procarbazine, lomustine and vincristine by targeting the above‐mentioned genetic alterations with hope to halt metastasis and invasion in high‐grade astrocytoma.^41^ Common molecular alterations in paediatric LGG and HGG are given in Table 4 along with recent clinical trials that target these alterations.

**TABLE 4:** Common molecular mutations in paediatric astrocytomas and current clinical trials targeting these alterations.^25^, ^63^, ^75^, ^76^, ^106^

Molecular marker	Grade 1	Grade 2	Grade 3	Grade 4	Features associated with the marker	Current clinical trials
*ATRX*	**✓**	**✓**	**✓**	**✓**	Associates with *IDH* and *TP53* mutations. Seen commonly in HGG	
*FGFR2*	**✓**	**✓**	**✓**	**✓**	This gene mutation shows mutual exclusivity with *IDH* and *EGFR* mutations	NCT03210714 NCT02885324
*HDAC*	**✓**	**✓**	**✓**	**✓**	Mainly in DIPG	NCT04264143 NCT03566199
*MAPK/BRAF*	**✓**	**✓**	**✓**	**✓**	Commonly undergoes mutation in LGG. It is a marker to distinguish between PA and GBM	*BRAFV600E* ‐ NCT01748149 ‐ NCT03975829 ‐ NCT05180825 ‐ NCT03919071 ‐ NCT04201457 *BRAFV600E/IDH1* and non*‐NF1*, non*‐TSC* ‐ NCT04576117 *BRAF* and *MEK1/2* ‐ NCT02684058 ‐ NCT03919071 *MAPK/ERK* ‐ NCT03363217 *MAPK/MEK1* and *CDK2/4* ‐ NCT03434262 *MEK1/2* ‐ NCT02285439 ‐ NCT04923126
*NF1*	**✓**	**✓**	**✓**	**✓**	Undergoes complete deletion in pilocytic astrocytoma. This deletion is seen as a cancer predisposition syndrome	NCT01553149
*PARP*	**✓**	**✓**	**✓**	**✓**	In LGG and HGG	NCT03749187 NCT03233204
*RB*	**✓**	**✓**	**✓**	**✓**	Exerts a significant effect on survival in all ages	NCT03355794 NCT03178032
*CDKN2A*	**✓**	**✓**			Mainly seen in LG astrocytomas. Usually undergoes homozygous deletions in tumours	
*P13K/mTOR*	**✓**		**✓**	**✓**	Rare in non‐aggressive tumours. Commonly seen mutated in GBM	NCT03213678 NCT02420613
*EZH2*		**✓**	**✓**	**✓**	Commonly seen in aggressive astrocytomas other solid tumours and rarely in the other forms.	NCT03155620 NCT03213665 NCT03605550
*IDH1/2*		**✓**	**✓**	**✓**	*IDH* mutations are rare in children <14 years old. Genes show hotspot mutations that are mutually exclusive. It is used to distinguish between PA and other grades	NCT04195555
*PDGFRA*		**✓**	**✓**	**✓**	Common in DIPG. It is the most frequent target of focal amplification	
*TP53*		**✓**	**✓**	**✓**	Commonly seen in aggressive astrocytomas and rarely in the other forms	
*ACVR1*			**✓**	**✓**	Seen in GBM and DIPG. Approximate mean survival is as low as 6 months	
*ADAMTS10*			**✓**	**✓**	Mainly in HGG especially in DIPG	NCT04295759
*DRD2*			**✓**	**✓**	Upregulated in HGG such as DIPG and DMG	NCT03416530
*EGFR*			**✓**	**✓**	*EGFR* genes undergo methylation in highly aggressive astrocytoma. Rarely seen in LGG	NCT03620032 NCT04532229 NCT02233049 NCT01884740
*H3.1*			**✓**	**✓**	Approximate mean survival rate is around 6 months with this mutation	*H3.3K27M*—NCT02960230
*MYC/MYCN*			**✓**	**✓**	Amplified in GBM and DIPG	
*PD‐1*			**✓**	**✓**	Mainly in HGG and DIPG	NCT03690869
*PTEN*			**✓**	**✓**	Rare in non‐aggressive tumours. Commonly seen mutated in GBM	

Abbreviations: *ACVR1*, activin receptor type‐1; *ATRX*, alpha‐thalassemia/mental retardation; *BRAF*, v‐raf murine sarcoma viral oncogene homologue B1; DIPG, diffuse intrinsic pontine gliomas; *DRD2*, dopamine receptor D2; *EGFR*, epidermal growth factor receptor; *FGFR2*, fibroblast growth factor receptor 2; GBM, glioblastoma multiforme; HDAC, histone deacetylase; X‐linked; HGG, high‐grade gliomas; *H31*, histone protein 31; *IDH*, isocitrate dehydrogenase; LGG, low‐grade gliomas; *MAPK*, mitogen‐activated protein kinases; *NF1*, neurofibromatosis type 1; PA, pilocytic astrocytoma; *PDGFRA*, platelet‐derived growth factor receptor alpha; *PTEN*, phosphatase and tensin homologue; *RB*, retinoblastoma; *TP53*, tumour protein 53.

## 10 RECENT CLINICAL ADVANCES IN EPIGENETIC ALTERATIONS

Epigenetics as defined by C. H. Waddington, is ‘the causal interactions between genes and their products, which brings the phenotype into being’ that has been modified because of ‘heritable changes in gene expression that occur independent of changes in the primary DNA sequence’. In simpler terms, epigenetics denotes the mitotically heritable alterations, which do not cause mutations in DNA sequences but have effects on the phenotype. Recent advances in understanding epigenetic alterations have opened new ways for developing therapies, which help restore or improve current conventional therapies against different paediatric and adult cancers.^109^ When it comes to pA, it is evident from the literature that major epigenetic changes in these tumours can be classified into three categories based on alterations in DNA methylation, chromatin remodelling and histone modification, with methylation changes being the most studied in pA (Table 5).^6^, ^110^ The epigenetic landscape of each tumour is unique and represents the characteristic methylation signatures corresponding to their molecular histology, which makes it an ideal criterion to inform treatment strategies as well as to better aid diagnostic guidelines.^111^ Since an accurate diagnosis of the different tumour subtypes is crucial to avoid inter‐observer variabilities and misdiagnosis, large‐scale attempts have been made to generalise the use of individual tumour‐based epigenetic profiling as a preliminary step in the clinical management of brain tumours. For example, a large‐scale cohort study done by Capper et al. yielded a comprehensive methylation‐based classification system for approximately 100 different brain tumours.^112^ The study also found that location‐specific epigenetic profiles exist for astrocytomas in paediatric patients and that they are reflective of the cell type these tumours originate from and proved the existence of three different methylation clusters concerning the location of the tumour. Based on these unique methylation profiles, the study generated an online classifier tool for classifying different tumour entities and reported that methylation‐based classification of paediatric brain tumours can help distinguish between malignant and less malignant neoplasms.^112^ Important markers of altered methylation in both paediatric and adult tumours include DNA‐5‐hydroxymethylcytosine (5hmC), levels of which increases with tumour progression, CpG island methylator phenotype (G‐CIMP), a hallmark prognostic marker of paediatric and adult tumours and *MGMT* promoter hypermethylation, observed in most gliomas and is an excellent indicator of TMZ resistance, especially in GBM.^113^ All these markers retain a distinct methylome profile making them excellent tools for targeted therapy.

**TABLE 5:** Common epigenetic alterations and their status in paediatric astrocytoma (pA).

Epigenetic alteration	Status	Pathogenesis on pA
*DNMT*	Upregulated through hypomethylation	Acts synergistically with EZH2 and promotes tumour progression^49^, ^119^
*EMP3*	Promoter‐associated hypermethylation	Impaired tumour suppression^120^
*EZH2*	Automethylation	Inhibits the activity of *PRC2* leading to higher level of histone methyltransferase activity^121^
*H3*	Acetylation, hyper/hypomethylation	Leads to extremely poor OS by conferring drug resistance and radiation resistance^114^
*HDAC*	Upregulated through hypomethylation	Leads to increased cell survival and metastasis^122^
*JARID1C (KDMC5)*	Hypomethylated	Promotes tumour progression and activation of oncogenes^123^
*LSD1*	Upregulated through hypermethylation	Increased proliferation and metastasis^124^
*NF1*	Biallelic gene inactivation through methylation	Mutations inactivate TSC complex and fails to inactivate mTOR pathway^83^
*PRC2*	Hypermethylated	Altered repressive histone modifications, chromatin alterations, downregulation of gene expression and impairs cell identity^125^, ^126^
*RANK*	Hypermethylated	Impaired immune response^127^
*SLIT2*	Hypomethylated	Increased malignant progression and correlated with poor survival and immunosuppression

Abbreviations: *DNMT*, DNA methyl transferase; *EMP3*, epithelial membrane protein 3; *HDAC*, histone deacetylase; *JARID1C/KDMC5I*, lysine‐specific demethylase 5C; *LSD1*, lysine‐specific histone demethylase 1A; *NF1*, neurofibromatosis type 1; OS, overall survival; *PRC2*, polycomb‐repressive complex 2; *RANK*, RANK‐ligand; *SLIT2*, slit guidance ligand 2; TSC, tuberous sclerosis complex.

Epigenetic events associated with histone *H3* mutations have gained considerable attention regarding their role in tumour initiation, maintenance and progression. Using epigenome‐wide methylation arrays and chromatin immunoprecipitation sequencing (ChIP‐Seq) techniques, common epigenetic alterations identified in pA include activating acetylation mutations of *H3 Lys27* (*H3*K*27*ac) and *H3K9*ac, activating methylation mutations of *H3K4*me3 and *H3K4*me2, hypermethylation of *H3.3* Gly34Arg/Val (*H3.3*‐G34R/V), which occurs on the histone tail shown to upregulate the expression of oncogene *MYCN* in pGBM and hyper/hypomethylation of *H3.1/3.3*‐K27M and *H3.3‐ATRX‐DAXX* (death‐domain‐associated protein) associated with chromatin remodelling pathways.^114^ Interestingly, two of the *H3* modifications, Lysine 27 and 36 were found to be central to the neurological location of the tumour with H3K27 mutations observed in tumours found in the thalamus, pons, spinal cord and cerebellum, whereas H3K36 mutations most prevalent in tumours found in the cerebral hemisphere.^115^

*TP53* differential methylation is another hallmark in pHGG. Studies have shown a pattern where low survival outcomes are linked with an overexpression of P53 even in the absence of *TP53* alterations. Upon further investigation into this remarkable feature, it was found that even in the absence of *TP53*, chromosomal alterations were facilitated by aberrant methylation of chromosome segments located on 17p. This suggested that the differential methylation of genes other than *TP53* on the 17p chromosome must be involved in the progression of pA such as in AA.^116^, ^117^

Some of the other common epigenetic mutations reported in pA consist of those associated with chromatin regulation. In pHGG such as GBM, an oncometabolite, 2‐hydroxy glutarate which accumulates due to the mutation of *IDH* gene, affects the structure of chromatin by altering epigenetic variables such as histone post‐translational modification and global DNA methylation. This impairment leads to the inhibition of ten‐eleven translocation (*TET*)‐mediated demethylation leading to hypermethylation in the cells and as a result, a drop in patient survival was observed.^118^ Other non‐recurrent epigenetic mutations found in pA include chromodomain helicase DNA‐binding proteins, lysine‐specific demethylase, polycomb repressive complex 2, histone deacetylase 2, histone‐lysine N‐methyltransferase enzyme (*EZH2* and *SET2*), histone demethylase (*JARID1C*) and *H3*K4 mono‐methyltransferase (*MLL2*).^114^ In addition, other methylation markers in pA include genes involved in *WNT* signalling pathway such as secreted frizzled related protein (SFRP) group of proteins, those involved in *STAT* signalling pathway such as *RASSF* (Ras association domain family member), those involved in cell migration, proliferation and apoptosis such as slit guidance ligand 2 (*SLIT2*), epithelial membrane protein 3 (*EMP3*) and receptor for RANK‐ligand (*RANK*), respectively.^113^, ^114^, ^116^

Taking these studies into account and numerous other large‐scale genomic studies on paediatric brain cancers, it is well established that epigenetic changes are key factors that facilitate genetic and molecular regulations that contribute to tumour initiation and progression. These tumour subtypes are highly variable therefore treatment strategies should consider the genetic and epigenetic signature of each of these tumour subtypes in order to produce effective therapeutics.

## 11 TARGETING EPIGENETIC ALTERATIONS: ALTERNATIVE DIAGNOSTIC AND THERAPEUTIC TARGETS

**FIGURE 5:**
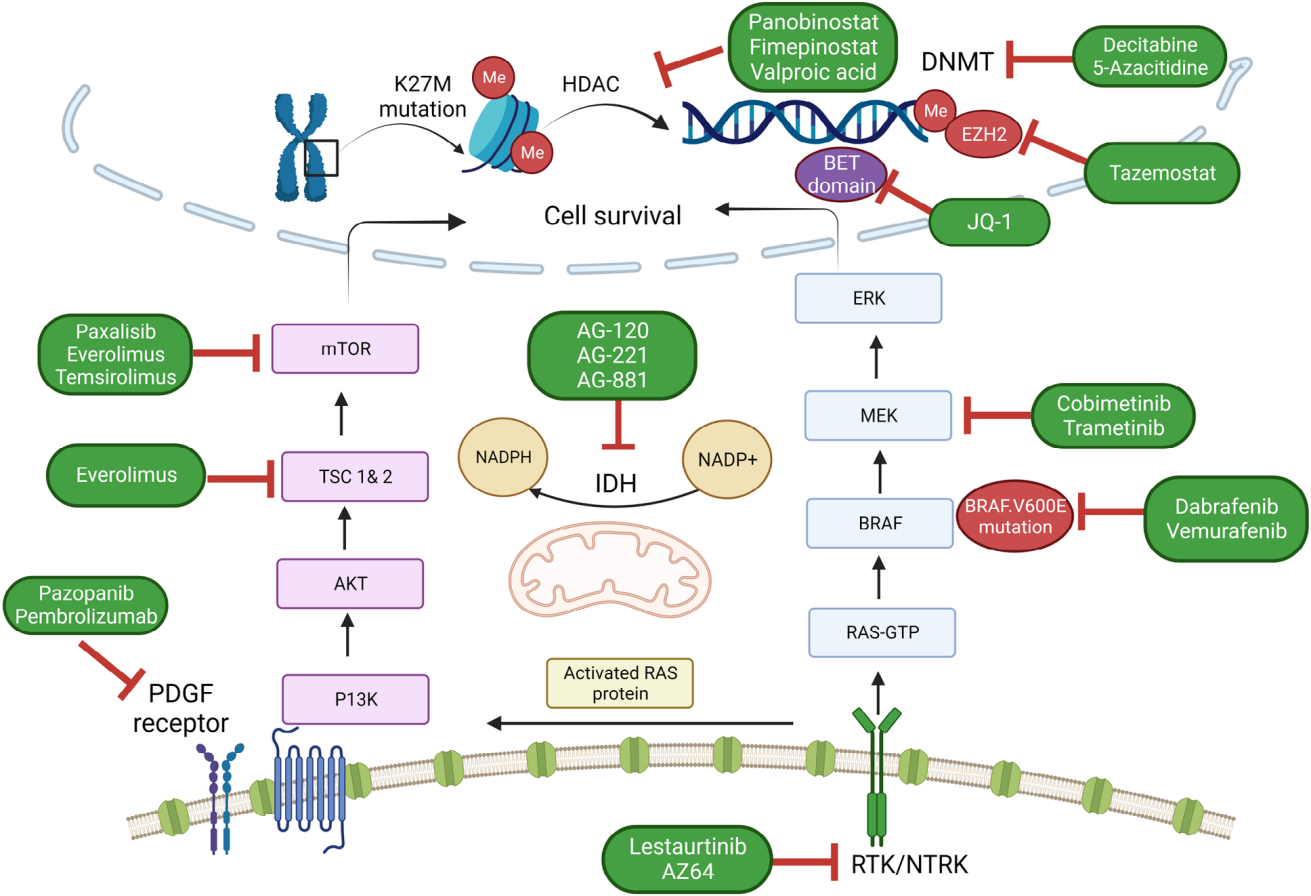
Important signalling pathways and their potential inhibitors involved in the epigenetic regulation of paediatric high‐ and low‐grade tumours (image created with Biorender.com). BET, bromodomain and extra‐terminal domain; DNMT, DNA methyl transferase; HDAC, histone deacetylase; IDH, mitochondrial isocitrate dehydrogenase; RTK/NTRK, receptor tyrosine kinase/neurotrophic tyrosine kinase receptor.

Increasing perceptions into the different epigenetic alterations that occur in a multitude of diseases have revealed to scientists that these changes can be utilised as potential markers for diagnosis, prognosis and therapy. Investigations into the mechanism of these alterations have accelerated the discovery of ‘epigenetic drugs’ targeted towards key enzymes, such as DNA methyl transferase (DNMT) and histone deacetylase (HDAC), known to be involved in imperative epigenetic pathways (Figure 5). Ever since the discovery of the cytotoxic/cancerostatic drug 5‐Azacytidin, a potent inhibitor of DNA methylation, and vorinostat, a potent pan‐HDAC inhibitor, research on potential epigenetic drugs for various cancers has made significant progress. However, research on these drugs in high grade brain tumours, let alone pHGG, remains under explored.

HDAC inhibitors (HDACi) are one of the most universally studied epigenetic drug classes in cancers. In vitro and in vivo studies on pHGG, primarily in DIPG, have shown substantial sensitivity in cells and reduced tumour growth in mice when treated with HDACi. Among the wide range of drugs tested in a high‐throughput drug screening study, panobinostat, a pan‐HDACi, demonstrated the highest therapeutic effects on DIPG cells and xenograft tumours, with decreased cell viability and tumour growth and increased mouse survival rates.^128^ This work has led to a phase 1 clinical trial (NCT02717455) which looked at the safety and doses of panobinostat in paediatric patients with recurrent/progressive DIPG and DMG. It was reported that panobinostat had limited tolerability and variable pharmacokinetics in the paediatric patients with DIPG/DMG and showed no clinical benefit between treatment groups. It was suggested that further treatment regimens, enhanced drug delivery methods and combination therapies should be considered in future clinical studies using panobinostat.^129^ Other clinical studies that use panobinostat include a phase 1 trial using Marizomib, a proteasome inhibitor in combination with panobinostat in pHGGs (NCT04341311) and phase 1/2 trials that are looking at the safety profile and drug tolerability of MTX110, a water soluble nanoformulation of panobinostat, in children newly diagnosed with DIPG (NCT03566199 and NCT04264143). Some other clinical trials using HDACi to treat pA that are currently underway/completed or in the recruiting phase are with vorinostat (class 1, 2 and 4 HDACi) alone or given together with RT (NCT01189266) or in combination with temsirolimus, a mTOR kinase inhibitor and RT (NCT02420613) and valproic acid (NCT00107458).^119^ Currently, there are four HDACi, panobinostat, vorinostat, romidepsin (class 1 HDACi) and belinostat (class 1 and 2 HDACi), approved by the FDA to be used in combination with conventional therapies for adult cancers,^130^ however as treatments for paediatric brain cancer, these inhibitors are still in phase I/II clinical trials.

Other epigenetic targets that are currently being tested in preclinical and phase 1 clinical studies as diagnostic biomarkers or therapeutic targets in paediatric brain cancers include DNMT, LSD1, lysine methyltransferase and bromodomain and extra‐terminal (BET) protein inhibitors.^131^ Of these, BET inhibitors used in combination with other molecular inhibitors such as tazemetostat (EZH2 inhibitor),^132^ CREB‐binding protein inhibitor^133^ or NOTCH pathway inhibitor MRK‐003^134^ have shown promising therapeutic outcomes in in vitro and in vivo pHGG models. Currently, a phase 1 clinical trial of BET inhibitors (BMS‐986158 and BMS‐986378) is underway to determine its safety and pharmacokinetic profile in children with primary or relapsed brain cancer (NCT03936465). Another interesting area of epigenetic therapy is their potential to be used as adjuvants for immunotherapy. Recent studies by Kailayangiri et al.^135^ and Mabe et al.^136^ have shown the ability of EZH2 inhibitors to enhance expression of ganglioside GD2 surface antigen in Ewing sarcoma and neuroblastoma cells, thereby increasing the efficacy of anti‐GD2 immunotherapies. Hence, it was proposed combining EZH2 inhibitors and anti‐GD2 immunotherapy may hold clinical benefits, not only for the studied childhood cancers, but also for other CNS tumours. Similarly, HDACi and DNMTi have shown to increase expression of tumour antigens, immune‐associated genes and in reversing epigenetic silencing of Th1‐type chemokines in adult cancer, and thus have strong potential to be used in combination with immunotherapy.^137^

Although there are a range of clinical trials testing different immunotherapies (chimeric antigen receptor T cells, checkpoint inhibitors, oncolytic virus and vaccines therapy) for paediatric brain cancers,^130^ only a limited number of clinical trials are studying the use of immunotherapies targeting epigenetic regulators. Currently, two early phase trials are testing the activity of vaccines against the H3.3 mutation present in pHGGs. NCT02960230 a phase 1/2 trial, currently active but not yet recruiting participants, is assessing the safety and therapeutic activity of a synthetic peptide vaccine targeting H3.3K27M antigen given alone or in combination with nivolumab (PD‐1 inhibitor) in children with DIPG or glioma. NCT04749641, a phase 1 trial, currently active and recruiting, is assessing the safety and tolerability of a neoantigen peptide vaccine targeting H3.3K27M mutation in DIPG via T‐cell activation.

## 12 CLINICAL PROSPECTS AND FUTURE DIRECTIONS

**FIGURE 6:**
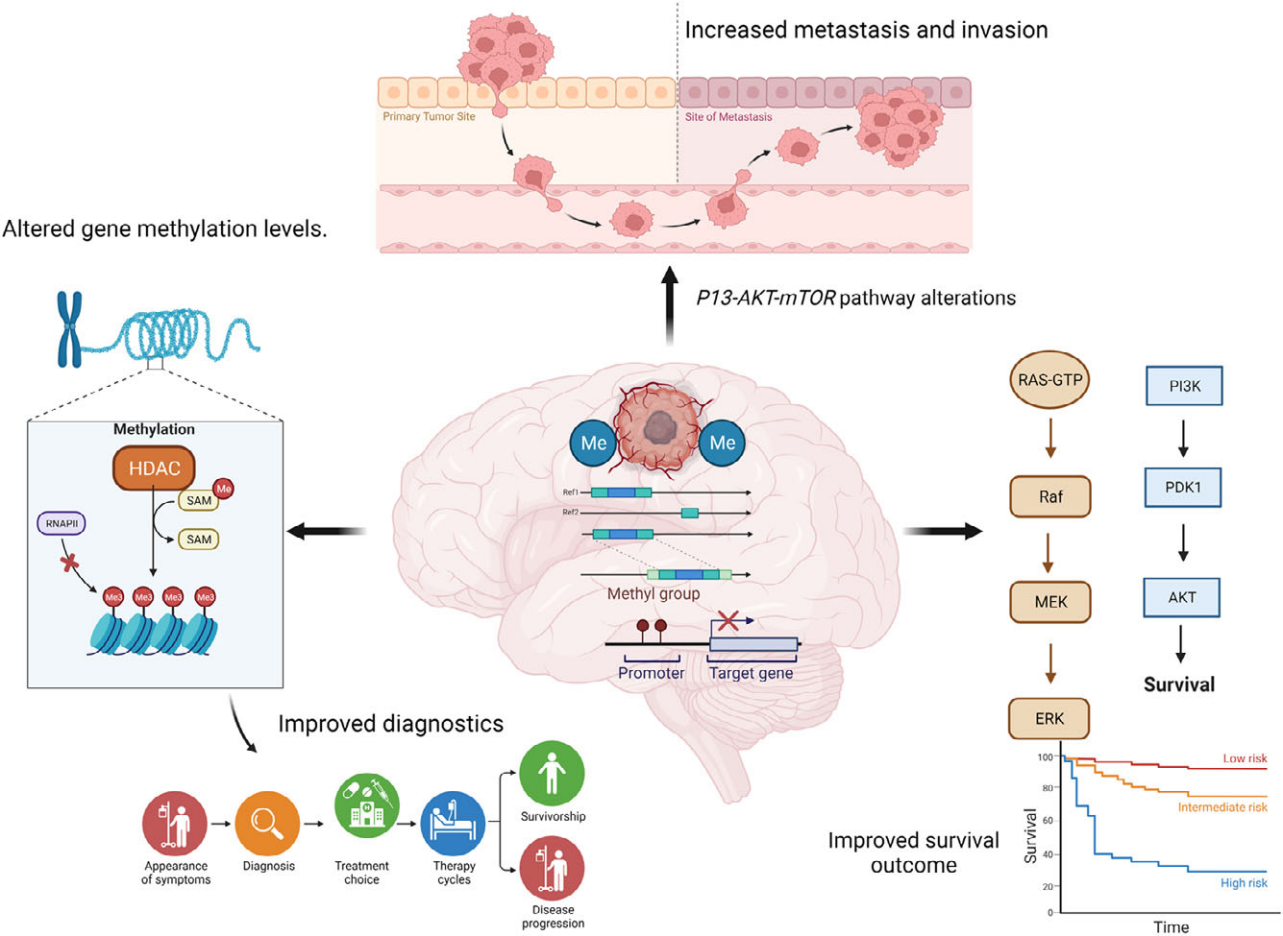
Importance of evaluating both genetic and epigenetic alterations when treating paediatric astrocytomas. Increased tumour metastasis and poor survival is linked to key genetic alterations including v‐raf murine sarcoma viral oncogene homologue B1 (BRAF), mitogen‐activated protein kinases (MAPK) and wingless‐related integration sites (WNT) pathways. Epigenetic deregulation in promoter regions of genes as well as mutations in histone deacetylases is also associated with tumour progression and metastasis. Hence, to ensure maximum therapeutic benefit for paediatric astrocytomas (pA) patients, multi‐omics analysis of the genome, transcriptome and epigenome must be considered when determining the best treatment regimen which will potentially improve survival outcomes for these patients.

While our understanding of molecular markers in paediatric high‐ and low‐grade astrocytomas has improved over time, the dependency of individual mutations on epigenetic factors are less understood. As research to date suggests, epigenetic drivers are extremely important in facilitating the pathology and progression of tumour in paediatric patients, hence incorporating these epigenetic and genetic biomarker profiles during diagnosis and therapy may hold significant therapeutic benefits. Clinical management of pA is still principally based on conventional treatment strategies such as surgery and chemotherapy. Such treatment approaches are based on established histo‐pathological analysis and classification based on molecular alterations and fail to consider factors such as DNA methylation or histone modifications when considering treatment regimens. Given the recent gain in attention in studying epigenetic markers in diagnosing diseases and predicting prognosis, recent clinical advances have started to show the therapeutic benefits of targeting these epigenetic alterations when used in combination with conventional chemotherapies. As summarised in Figure 5, certain epigenetic targeting drugs are currently in the clinical stage and used to treat different adult cancers however its clinical use in treating paediatric cancers, in particularly pA, is significantly lacking. Translational blocks that have hindered the clinical use of epigenetic targeted drugs include their ability to induce drug resistance in cancer cells, inability to effectively penetrate the blood brain barrier and their toxic effects on non‐cancerous tissues that may also express epigenetic regulators. Targeting strategies such as the use of antibodies or aptamers may be promising avenues that need to be further studied in order to optimise systemic targeted delivery of these drugs. Therefore, continued research and clinical trials in this field is essential to unlock the full potential of epigenetic targeting in paediatric oncology. Combining both genetic and epigenetic biomarker analysis and integrating this information into clinical practice holds great promise for improving diagnostics, treatment responses, survival outcomes and importantly quality of life of paediatric patients battling these aggressive forms of brain cancers (Figure 6).

## AUTHOR CONTRIBUTIONS

*Conceptualisation*: Dona A. Johns, Richard J. Williams, Craig M. Smith, Pavani P. Nadaminti and Rasika M. Samarasinghe. *Writing—original draft preparation*: Dona A. Johns and Rasika M. Samarasinghe share equal contribution. *Review and editing*: Dona A. Johns, Richard J. Williams, Craig M. Smith, Pavani P. Nadaminti and Rasika M. Samarasinghe. All authors have read and agreed to the published version of the manuscript.

## CONFLICT OF INTEREST STATEMENT

The authors declare they have no conflicts of interest.

## ETHICS STATEMENT

Not applicable.

## Data Availability

Not applicable.
